# Design, Synthesis and Biological Evaluation of 4-Benzamidobenzoic Acid Hydrazide Derivatives as Novel Soluble Epoxide Hydrolase Inhibitors 

**Published:** 2014

**Authors:** Elham Rezaee Zavareh, Mahdi Hedayati, Laleh Hoghooghi Rad, Soraya Shahhosseini, Mehrdad Faizi, Sayyed Abbas Tabatabai

**Affiliations:** a *Department of Pharmaceutical Chemistry, School of Pharmacy, Shahid Beheshti University of Medical Sciences, Tehran, Iran.*; b *Cellular and Molecular Research Center, Research Institute for Endocrine Sciences, Shahid Beheshti University of Medical Sciences, Tehran, Iran.*; c *Department of Pharmacology and Toxicology, School of Pharmacy, Shahid Beheshti University of Medical Sciences, Tehran, Iran. *

**Keywords:** Synthesis, Docking, Benzamidobenzoic acid hydrazide, Soluble epoxide hydrolase, Physical properties

## Abstract

Inhibitors of soluble epoxide hydrolase (sEH) represent one of the novel pharmaceutical approaches for treating hypertension, vascular inflammation, pain and other cardiovascular related diseases. Most of the potent sEH inhibitors reported in literature often suffer from poor solubility and bioavailability. Toward improving pharmacokinetic profile beside favorable potency, two series of 4-benzamidobenzoic acid hydrazide derivatives with hydrazide group as a novel secondary pharmacophore against sEH enzyme were developed. The designed compounds were synthesized in acceptable yield and their in vitro assay was determined. Most of the synthesized compounds have appropriate physical properties and exhibited considerable *in-vitro *sEH inhibitory activity in comparison with 12-(3-Adamantan-1-yl-ureido)- dodecanoicacid (AUDA), a potent urea-based sEH inhibitor. 4-(2-(4-(4-chlorobenzamido) benzoyl)hydrazinyl)-4-oxobutanoic acid 6c was found to be the most potent inhibitor with inhibitory activity of 72% targeting sEH enzyme.

## Introduction

Human soluble epoxide hydrolase enzyme converts epoxyeicosatrienoic acids (EETs) to their corresponding hydrated products by catalyzing the addition of water to the epoxide moiety (1, 2). EETs have a wide range of physiological effects. *e. g*., increase sodium renal excretion, relax vascular conduit and dilate renal afferent arterioles and coronary resistance vessels (3, 4). In addition, EETs modulate leukocyte adhesion, platelet aggregation, vascular smooth muscle cell migration and thrombolysis in preclinical animal models (5). Therefore, inhibition of sEH might be a promising new treatment in hypertension, vascular inflammation, pain and other cardiovascular related diseases (3-6). 

**Figure 1:**
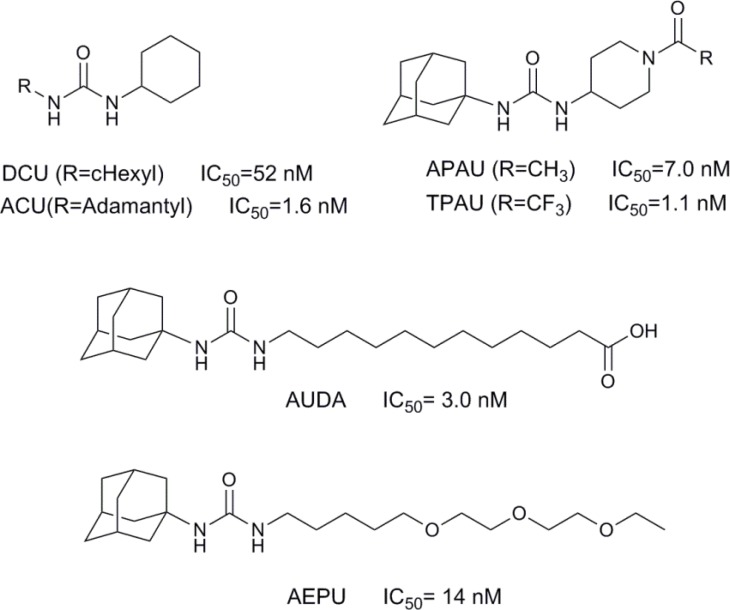
Chemical structures of known sEH inhibitors

The previous studies revealed the hydrolase catalytic pocket of sEH consists of two tyrosine and an aspartate residues which act essential role in epoxide ring opening (6). It has been recognized that amide or urea groups fit well in the hydrolase catalytic pocket to interact with mentioned residues. Specifically, the carbonyl oxygen of the amide or urea is engaged in a hydrogen bond interaction with tyrosine and the N–H moiety acts as a hydrogen bond donor to aspartate. Therefore, various urea and amide analogues have been developed as reversible sEH inhibitors (2-3, 7). Urea, carbamate, and amide compounds substituted with hydrophobic groups are potent and stable sEH inhibitors. However, poor physical properties of these compounds, such as low solubility and high melting points, lead to limited *in-vivo *availability (8). Solubility and bioavailability improved with the addition of a polar functional group on specific positions of one of the urea or amide moiety (9-11). Therefore, 12-(3-adamantan-1-ylureido) dodecanoic acid (AUDA) and 1-adamantan-1-yl-3-{5-[2-(ethoxyethoxy) ethoxy]pentyl} urea (AEPU) were developed (Figure 1) (12).

**Figure 2:**
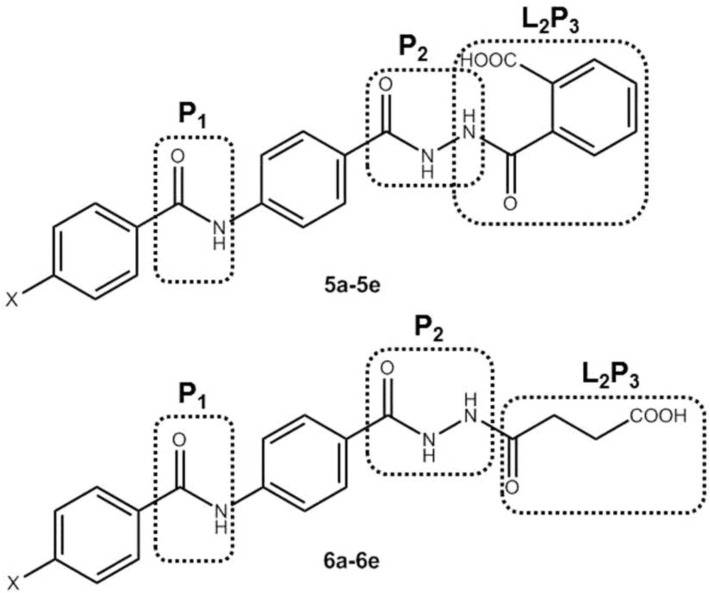
General scaffold of the synthesized analogues

According to the pharmacophore model suggested for sEH inhibitors (1, 13, 14), we designed, synthesized and biologically evaluated two series of 4-benzamidobenzoic acid hydrazide derivatives as novel soluble epoxide hydrolase inhibitors (Figure 2). The amide group in the represented structures is considered as the primary pharmacophore (P1) and the hydrazide group is the secondary pharmacophore (P2). Phenyl ring joins P1 and P2 together as a lipophilic spacer. Oxobutanoic acid and carbonylbenzoic acid moieties play the role of terminal pharmacophore (L2/P3). Phenyl ring with various hydrophobic or hydrophilic substitutes in the R position were added to the main structure.

## Experimental

*Chemistry*

**Figure 3:**
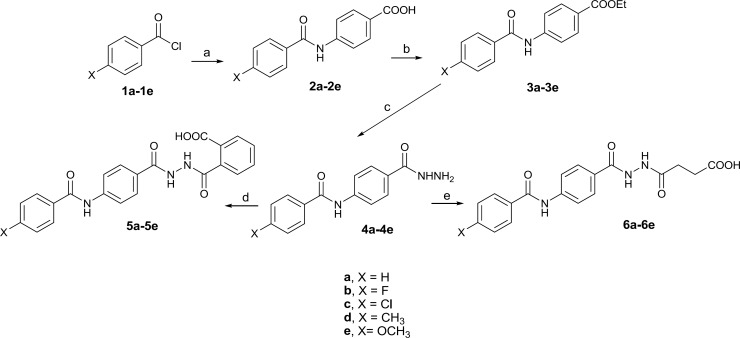
Schematic representation of synthesis of the designed compounds. Reagents and conditions: (a) 4-aminobenzoic acid, anhydrous Na2CO3, THF, rt, 6-12 h, 60-85%; (b) H2SO4, EtOH, reflux, 24 h, 58-65%; (c) NH2NH2.H2O, EtOH, rt, 12 h, 70-80%; (d) phthalic anhydrides, toluene, rt, 24 h, 65-70%; (e) succinic anhydride, toluene, rt, 18 h, 72-80%.

All laboratory grade reagents were obtained commercially from Aldrich or Merck Company. The reactions were monitored by thin layer chromatography (TLC) performed on commercially available Merck precoated plates (silica gel 60 F254, 0.25 mm). The structures of the synthesized compounds were confirmed by IR, LC/MS and 1HNMR. Perkin Elmer 843 IR and Agilent 6410 (QQQ) LC/MS were used to obtain IR and Mass spectra respectively.1HNMR spectra were recorded on a Bruker advance II (500 MHz) spectrophotometer using [D6] DMSO as a solvent. Water solubility was determined experimentally in 1.0 mL of sodium phosphate buffer (0.1 M, pH 7.4) at 25 ± 1 °C (9, 15). The logP (octanol/water partition coefficient (P)) values were calculated by Crippen’s method using CS ChemBioDraw Ultra version 12.0 software and melting points were taken on a Electrothermal 9100 apparatus and are uncorrected. The designed compounds were synthesized as shown in Figure 3.

*General procedure for the preparation of 4-(4-substitutedbenzamido)benzoic acid (2a-2e)*

A solution of 4-aminobenzoic acid (1.68 mmol) and para substituted benzoylchlorides (1.68 mmol) in dry THF, in presence of anhydrous Na2CO3 (1.68 mmol) was stirred at room temperature for 6-12 h. The solvent was evaporated and the precipitate was washed with water and recrystallized from ethanol 96% to give final products.

*4-benzamidobenzoic acid (2a)*

Yield: 85%; white crystalline powder; mp: 150-151 °C, IR (KBr): υ (cm-1) 2887-3045 (OH), 3340 (NH), 1675, 1650 (C=O); LC-MS (ESI) m/z = 242 (M+1, 25%), 264 (M+23, 100%).

*4-(4-fluorobenzamido)benzoic acid (2b)*

Yield: 80%; white crystalline powder; mp: 155-156 °C, IR (KBr): υ (cm-1) 2857-3125 (OH), 3379 (NH), 1696, 1685 (C=O); LC-MS (ESI) m/z = 260 (M+1, 30%), 282 (M+23, 100%).

*4-(4-chlorobenzamido)benzoic acid (2c)*

Yield: 75%; white crystalline powder; mp: 138-140 °C, IR (KBr): υ (cm-1) 2730-3045 (OH), 3320 (NH), 1695, 1670 (C=O); LC-MS (ESI) m/z = 276 (M+1, 60%), 298 (M+23, 100%).

*4-(4-methylbenzamido)benzoic acid (2d)*

Yield: 80%; white crystalline powder; mp: 145-147 °C, IR (KBr): υ (cm-1) 2630-3145 (OH), 3340 (NH), 1670, 1690 (C=O); LC-MS (ESI) m/z = 256 (M+1, 30%), 278 (M+23, 100%).

*4-(4-methoxybenzamido)benzoic acid (2e)*

Yield: 60%; cream crystalline powder; mp: 150-152 °C, IR (KBr): υ (cm-1) 2807-3015 (OH), 3320 (NH), 1695, 1660 (C=O); LC-MS (ESI) m/z = 272 (M+1, 40%), 294 (M+23, 100%).

*General procedure for the preparation of Ethyl 4-(4-substitutedbenzamido)benzoate (3a-3e)*

8.06 mmol of 2 was dissolved in ethanol (15 mL) and concentrated sulfuric acid (0.5 mL) was added. The solution was refluxed for 24 h. Then, ethanol was evaporated and the remnant was alkalized after being cooled in the ice bath with NaOH 20% and extracted with diethyl ether. The diethyl ether phase was washed first with aqueous NaOH 20% and water and dried with anhydrous sodium sulfate and evaporated.

*Ethyl 4-benzamidobenzoate (3a)*

Yield: 60%; white crystalline powder; mp: 95-97, IR (KBr): υ (cm-1) 3340 (NH), 1720, 1670 (C=O); LC-MS (ESI) m/z = 270 (M+1, 100%), 292 (M+23, 30%).

*Ethyl 4-(4-fluorobenzamido)benzoate (3b)*

Yield: 65%; white crystalline powder; mp: 90-92 °C, IR (KBr): υ (cm-1) 3330 (NH), 1715, 1670 (C=O); LC-MS (ESI) m/z = 288 (M+1, 100%), 310 (M+23, 55%).

*Ethyl 4-(4-chlorobenzamido)benzoate (3c)*

Yield: 63%; white crystalline powder; mp: 96-97 °C, IR (KBr): υ (cm-1) 3315 (NH), 1690, 1730 (C=O); LC-MS (ESI) m/z = 304 (M+1, 100%).

*Ethyl 4-(4-methylbenzamido) benzoate (3d)*

Yield: 65%; white crystalline powder; mp: 99-100 °C, IR (KBr): υ (cm-1) 3340 (NH), 1730, 1680 (C=O); LC-MS (ESI) m/z = 284 (M+1, 50%), 306 (M+23, 100%). *Ethyl 4-(4-methoxybenzamido)benzoate (3e)*

Yield: 58%; cream crystalline powder; mp: 105-106 °C, IR (KBr): υ (cm-1) 3330 (NH), 1715, 1690 (C=O); LC-MS (ESI) m/z = 300 (M+1, 100%).

*General procedure for the preparation of 4-substituted-N-(4-hydrazinecarbonyl) phenyl)benzamide (4a-4e)*

7.24 mmol of 3 and 10 mL hydrazine hydrate (200 mmol) were added to 10 mL ethanol. The mixture was stirred for 12 h at room temperature. Afterward, the solvent was evaporated and white precipitates washed with diethyl ether, and recrystallized from a mixture of ethanol and a few drops of water.

*N-(4-(hydrazinecarbonyl)phenyl)benzamide (4a)*

Yield: 80%; white crystalline powder; mp: 120-122 °C, IR (KBr): υ (cm-1) 3397, 3307 (NH), 1647 (C=O); LC-MS (ESI) m/z = 256 (M+1, 100%).

*4-Fluoro-N-(4-hydrazinecarbonyl)phenyl)benzamide (4b)*

Yield: 70%; white crystalline powder; mp: 125-126 °C, IR (KBr): υ (cm-1) 3336, 3317 (NH), 1659 (C=O); LC-MS (ESI) m/z = 274 (M+1, 100%).

*4-Chloro-N-(4-hydrazinecarbonyl)phenyl)benzamide (4c)*

Yield: 75%; white crystalline powder; mp: 127-129 °C, IR (KBr): υ (cm-1) 3340, 3327 (NH), 1670 (C=O); LC-MS (ESI) m/z = 290 (M+1, 100%).

*4-Methyl-N-(4-hydrazinecarbonyl)phenyl) benzamide (4d)*

Yield: 80%; white crystalline powder; mp: 120-121 °C, IR (KBr): υ (cm-1) 3336, 3307 (NH), 1659 (C=O); LC-MS (ESI) m/z = 270 (M+1, 100%).

*4-Methoxy-N-(4-hydrazinecarbonyl) phenyl)benzamide (4e)*

Yield: 70%; white crystalline powder; mp: 130-131 °C, IR (KBr): υ (cm-1) 3320, 3307 (NH), 1680 (C=O); LC-MS (ESI) m/z = 286 (M+1, 100%).

*General procedure for the preparation of 2-(2-(4-(4-substitutedbenzamido)benzoyl)hydrazinyl)carbonylbenzoic acid (5a-5e )*

A solution of 4 (1.68 mmol) and phtalic anhydrides (1.68 mmol) in dry toluene was stirred at room temperature for overnight. The solvent was evaporated and the precipitate was washed with water and recrystallized from ethanol 96%.

*2-(2-(4-benzamido)benzoyl) hydrazinylcarbonylbenzoic acid (5a)*

Yield: 70%; white crystalline powder; mp: 170 °C, IR (KBr): υ (cm-1) 2665-3315 (OH), 3268, 3231 (NH), 1694, 1666 (C=O); LC-MS (ESI) m/z = 404 (M+1, 100%); 1HNMR (DMSO/500 MHz): 7.57 (6H, m, H3, H4, H5-benzamido, H3, H4, H5-benzoicacid), 7.70 (1H, d, H6-benzoic acid, *J *= 8.0 Hz), 7.90 (2H, d, H3, H5-benzoyl, *J *= 8.0 Hz), 7.95 (2H, d, H2, H6-benzoyl, *J *= 8.0 Hz), 8.06 (2H, dd, H2, H6-benzamido, *J *= 8.0, 3.5 Hz), 10.35 (3H, s, NH), 12.00 (1H, br s, COOH).

*2-(2-(4-(4-fluorobenzamido)benzoyl)hydrazinyl)carbonylbenzoic acid (5b)*

Yield: 65%; white crystalline powder; mp: 172 °C, IR (KBr): υ (cm-1) 2657-3330 (OH), 3235, 3224 (NH), 1710, 1667 (C=O); LC-MS (ESI) m/z = 422 (M+1, 20%), 444 (M+23, 100%); 1HNMR (DMSO/500 MHz): 7.34 (2H, t, H3, H5-benzamido, *J *= 8.7 Hz), 7.58 (3H, m, H3, H4, H5-benzoicacid), 7.74 (1H, d, H6-benzoic acid, *J *= 8.5 Hz), 7.90 (2H, d, H3, H5-benzoyl, *J *= 8.5 Hz), 7.95 (2H, d, H2, H6-benzoyl, *J *= 8.5 Hz), 8.06 (2H, dd, H2, H6-benzamido, *J *= 8.7, 2.5 Hz), 10.35 (1H, s, NH), 10.45 (2H, br s, NH), 12.01 (1H, br s, COOH).

*2-(2-(4-(4-chlorobenzamido)benzoyl)hydrazinyl)carbonylbenzoic acid (5c)*

Yield: 70%; white crystalline powder; mp: 173 °C, IR (KBr): υ (cm-1) 2673-3270 (OH), 3250, 2865 (NH), 1677, 1657 (C=O); LC-MS (ESI) m/z = 438 (M+1, 100%); 1HNMR (DMSO/500 MHz): 7.14 (2H, d, H3, H5-benzamido, *J *= 8.7 Hz), 7.55 (3H, m, H3, H4, H5-benzoicacid), 7.74 (1H, d, H6-benzoic acid, *J *= 8.7 Hz), 7.80 (2H, d, H3, H5-benzoyl, *J *= 8.5 Hz), 7.90 (2H, d, H2, H6-benzoyl, *J *= 8.5 Hz), 8.00 (2H, d, H2, H6-benzamido, *J *= 8.7 Hz), 10.05 (1H, s, NH), 10.35 (2H, br s, NH), 12.20 (1H, br s, COOH).

*2-(2-(4-(4-methylbenzamido)benzoyl)hydrazinyl)carbonylbenzoic acid (5d)*

Yield: 65%; white crystalline powder; mp: 174 °C, IR (KBr): υ (cm-1) 2859-3400 (OH), 3308, 3227 (NH), 1700, 1680 (C=O); LC-MS (ESI) m/z = 418 (M+1, 100%), 440 (M+23, 60%); 1HNMR (DMSO/500 MHz): 2.39 (3H, s, CH3), 7.04 (2H, d, H3, H5-benzamido, *J *= 8.1 Hz), 7.25 (3H, m, H3, H4, H5-benzoicacid), 7.44 (1H, d, H6-benzoic acid, *J *= 8.7 Hz), 7.70 (2H, d, H3, H5-benzoyl, *J *= 8.0 Hz), 7.80 (2H, d, H2, H6-benzoyl, *J *= 8.0 Hz), 7.90 (2H, d, H2, H6-benzamido, *J *= 8.1 Hz), 10.25 (3H, s, NH), 12.20 (1H, br s, COOH).

*2-(2-(4-(4-methoxybenzamido)benzoyl)hydrazinyl)carbonylbenzoic acid (5e)*

Yield: 68%; white crystalline powder; mp: 170 °C, IR (KBr): υ (cm-1) 2940-3492 (OH), 3318, 3228 (NH), 1712, 1643 (C=O); LC-MS (ESI) m/z = 434 (M+1, 100%), 456 (M+23, 80%); 1HNMR (DMSO/500 MHz): 3.84 (3H, s, OCH3), 6.95 (2H, d, H3, H5-benzamido, *J *= Rezaee zavareh E *et al. */ IJPR (2014), 13 (supplement): 51-59 56 

8.1 Hz), 7.05 (3H, m, H3, H4, H5-benzoicacid), 7.20 (1H, d, H6-benzoic acid, *J *= 8.7 Hz), 7.30 (2H, d, H3, H5-benzoyl, *J *= 8.5 Hz), 7.60 (2H, d, H2, H6-benzoyl, *J *= 8.5 Hz), 7.80 (2H, d, H2, H6-benzamido, *J *= 8.1 Hz), 10.00 (3H, s, NH), 12.00 (1H, br s, COOH).

*General procedure for the preparation of 4-(2-(4-(4-substitutedbenzamido)benzoyl)hydrazinyl)-4-oxobutanoic acid (6a-6e)*

A solution of 4 (1.68 mmol) and succinic anhydride (1.68 mmol) in dry toluene was stirred at room temperature for 18 h. The solvent was evaporated and the precipitate was washed with water and recrystallized from methanol.

*4-(2-(4-benzamidobenzoyl) hydrazinyl)-4-oxobutanoic acid (6a)*

Yield: 80%; white crystalline powder; mp: 172 °C, IR (KBr): υ (cm-1) 2890-3402 (OH), 3235, 3021 (NH), 1684, 1667 (C=O); LC-MS (ESI) m/z = 356 (M+1, 100%), 378 (M+23, 50%); 1HNMR (DMSO/500 MHz): 2.40 (4H, s, CH2CH2), 7.57 (3H, m, H3, H4, H5-benzamido), 7.95 (4H, m, H2, H3, H5, H6-benzoyl), 8.27 (2H, dd, H2, H6-benzamido, *J *= 8.7, 3.5 Hz), 10.18 (3H, s, NH), 12.10 (1H, br s, COOH).

*4-(2-(4-(4-fluorobenzamido)benzoyl)hydrazinyl)-4-oxobutanoic acid (6b)*

Yield: 72%; white crystalline powder; mp: 170 °C, IR (KBr): υ (cm-1) 2936-3373 (OH), 3306, 3262 (NH), 1685, 1649 (C=O); LC-MS (ESI) m/z = 374 (M+1, 100%), 396 (M+23, 90%); 1HNMR (DMSO/500 MHz): 2.48 (4H, s, CH2CH2), 7.37 (2H, t, H3, H5-benzamido, *J *= 8.7 Hz), 7.90 (4H, m, H2, H3, H5, H6-benzoyl), 8.07 (2H, dd, H2, H6-benzamido, *J *= 8.7, 2.5 Hz), 10.18 (1H, s, NH), 10.44 (1H, s, NH), 10.57 (1H, s, NH), 12.10 (1H, br s, COOH).

*4-(2-(4-(4-chlorobenzamido)benzoyl)hydrazinyl)-4-oxobutanoic acid (6c)*

Yield: 75%; white crystalline powder; mp: 168 °C, IR (KBr): υ (cm-1) 2965-3492 (OH), 3297, 3043 (NH), 1686, 1644 (C=O); LC-MS (ESI) m/z = 390 (M+1, 100%); 1HNMR (DMSO/500 MHz): 2.38 (4H, s, CH2CH2), 7.27 (2H, d, H3, H5-benzamido, *J *= 8.7 Hz), 7.80 (4H, m, H2, H3, H5, H6-benzoyl), 8.00 (2H, d, H2, H6-benzamido, *J *= 8.7 Hz), 10.28 (1H, s, NH), 10.40 (1H, s, NH), 10.57 (1H, s, NH), 12.10 (1H, br s, COOH).

*4-(2-(4-(4-methylbenzamido)benzoyl)hydrazinyl)-4-oxobutanoic acid (6d)*

Yield: 80%; white crystalline powder; mp: 160 °C, IR (KBr): υ (cm-1) 2942-3501 (OH), 3312, 3273 (NH), 1702, 1661 (C=O); LC-MS (ESI) m/z = 370 (M+1, 100%); 1HNMR (DMSO/500 MHz): 2.39 (3H, s, CH3), 2.48 (4H, s, CH2CH2), 7.07 (2H, d, H3, H5-benzamido, *J *= 8.7 Hz), 7.50 (4H, m, H2, H3, H5, H6-benzoyl), 7.95 (2H, d, H2, H6-benzamido, *J *= 8.7 Hz), 10.20 (3H, s, NH), 12.00 (1H, br s, COOH).

*4-(2-(4-(4-methoxybenzamido)benzoyl)hydrazinyl)-4-oxobutanoic acid (6e)*

Yield: 72%; white crystalline powder; mp: 165 °C, IR (KBr): υ (cm-1) 2981-3513 (OH), 3323, 3284 (NH), 1709, 1667 (C=O); LC-MS (ESI) m/z = 386 (M+1, 20%), 408 (M+23, 100%); 1HNMR (DMSO/500 MHz): 2.38 (4H, s, CH2CH2), 3.84 (3H, s, OCH3), 7.00 (2H, d, H3, H5-benzamido, *J *= 8.3 Hz), 7.30 (4H, m, H2, H3, H5, H6-benzoyl), 7.75 (2H, d, H2, H6-benzamido, *J *= 8.3 Hz), 10.70 (3H, s, NH), 12.00 (1H, br s, COOH).

*Docking studies*

The high resolution crystal structure of sEH (PDB code: 3ANS) complexed with 4-cyano-N-[(1S, 2R)-2-phenylcyclopropyl]benzamide was retrieved from RCSB Protein Data Bank. The structures of compounds were investigated using the Lamarckian genetic algorithm search method implemented in AutoDock 4.0 software. The receptors were kept rigid, and ligands were allowed to be flexible. Polar hydrogens and Kollman united atom partial charges were added to the individual protein atoms. Each structure was energy minimized under MM+ method in HyperChem8 software and converted to pdbqt format file using AutoDockTools 4.0 version1.5.4. A docking grid box was built with 40, 40 and 40 points in 25.8460, 24.0730 and 114.8150 directions in the catalytic site of protein and the number of generations and maximum number of energy evaluations was set to 100 and 2,700,000, respectively. Docking results were clustered with a root mean square deviation (RMSD) of 0.5 Å and evaluated by Pymol software.

*In-vitro biological activity*

Biological evaluation was performed by Cayman fluorescence-based human soluble epoxide hydrolase assay kit (item number 10011671). The enzyme and inhibitors were incubated for 15 min in 25 mM Bis-Tris/HCl buffer (200 μL; pH 7.0) at 30 °C. 3-phenyl-cyano (6-methoxy-2-naphthalenyl) methyl ester-2-oxiraneacetic acid (PHOME) was used as the substrate for assay. The reference inhibitor for assay is AUDA, one of the most effective inhibitors of sEH with 50% inhibitory activity in 1 nM concentration. Test samples were dissolved in DMSO and tested in 1 nM concentration in duplicate to the determination of the inhibitory activity.

## Result and Discussion

**Figure 4:**
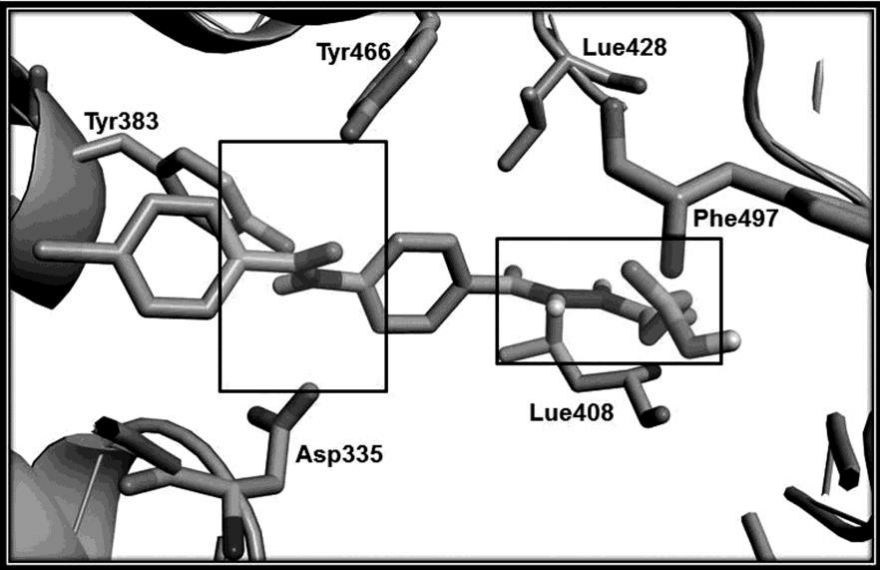
The minimized docking model of compound 6c in human sEH. The windows show the location of the amide (P1) and hydrazide (P2) groups in active site

The designed compounds were synthesized in good yield according to Figure 3. Compounds 2a-2e was prepared from the reaction of 4-aminobenzoic acid with appropriate benzoyl chlorides (16). Hydrazides 4a-4e was synthesized through esterification of the corresponding acids followed by treatment with hydrazine hydrate (17, 18). The final products 5a-5e and 6a-6e were obtained from the reaction of proper hydrazides with phthalic and succinic anhydride respectively. Molecular structures of the synthesized compounds were confirmed by IR, Mass, and 1HNMR spectroscopy. The unusual result was observed in 1HNMR data of the butanoic acid analogues (6a-6e). Two vicinal methylene groups of the butanoyl moiety were singlet. It seems that they have an exactly equal chemical shift accidentally so they couldn’t split each other. Docking study on designed sEH inhibitors confirms that the analogues fit in the hydrolase catalytic pocket of X-ray crystal structure of sEH. As shown in Figure 4, it is obvious that 4-(2-(4-(4-chlorobenzamido) benzoyl)hydrazinyl)-4-oxobutanoic acid (6c) in the active site pocket have a suitable distance from the three amino acids of Tyr383, Tyr466 and Asp335 for effective hydrogen bonding and additional hydrogen bonds could be formed between this compound and Phe497, Lue408 and Lue428 of the catalytic pocket.

According to Table 1, most of the synthesized compounds show a considerable inhibitory activity in 1 nM concentration in comparison with inhibitory activity of AUDA, the potent urea-based inhibitor with 50 % inhibitory ratio in the equal concentration. The most potent compounds in both series are those with chloro substituent on the 4-position of the phenyl ring (5c and 6c) with inhibitory activity of 47% and 72% respectively. Generally the butanoic acid analogues (6a-6e) were found to be more potent than the corresponding benzoic acid derivatives (5a-5e) and also have a better water solubility property. Since all of the synthesized compounds have carboxylic moiety, the solubility of these compounds could be improved more by synthesis of the corresponding carboxylate salts. It seems the butanoic acid derivatives could be appropriate candidates for more investigation about new therapeutic agents due to acceptable solubility with proper inhibitory activity. In conclusion, new 4-benzamidobenzoic acid hydrazide derivatives against soluble epoxide hydrolase enzyme were investigated. According to the inhibitory activities and solubility properties the designed structures might be valuable lead scaffold to development of the potent inhibitors with improved pharmacokinetic properties.

**Table 1:** Inhibitory activityof thesynthesized derivatives andthe related physical properties

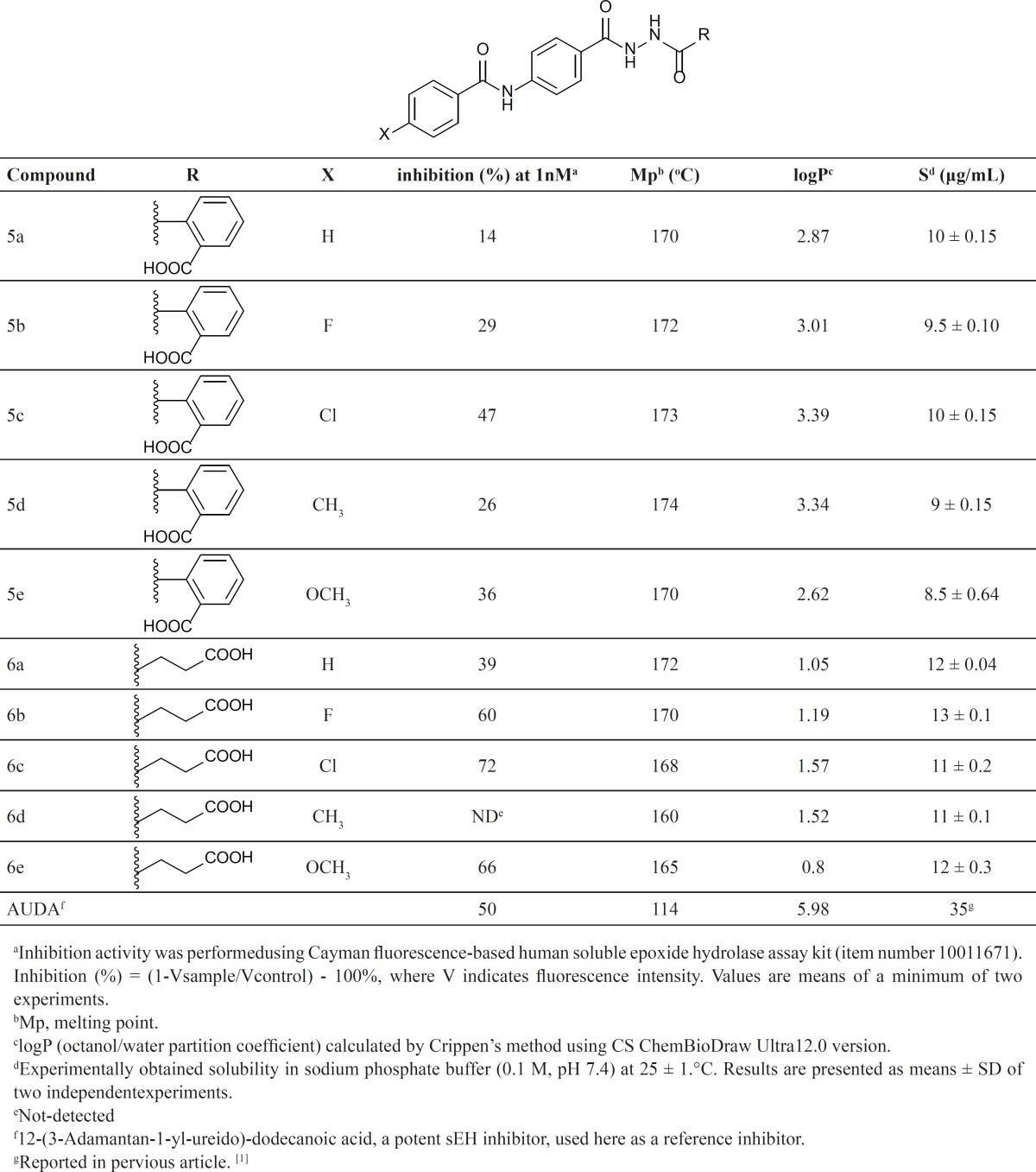

^a^Inhibition activity was performedusing Cayman fluorescence-based human soluble epoxide hydrolase assay kit (item number 10011671). Inhibition (%) = (1-Vsample/Vcontrol) - 100%, where V indicates fluorescence intensity. Values are means of a minimum of two experiments.

^b^Mp, melting point.

^c^logP (octanol/water partition coefficient) calculated by Crippen’s method using CS ChemBioDraw Ultra12.0 version.

^d^Experimentally obtained solubility in sodium phosphate buffer (0.1 M, pH 7.4) at 25 ± 1.°C. Results are presented as means ± SD of two independentexperiments.

^e^Not-detected

^f^12-(3-Adamantan-1-yl-ureido)-dodecanoic acid, a potent sEH inhibitor, used here as a reference inhibitor.

^g^Reported in pervious article. [1]
